# Supplementary material for: Long-read sequencing maps transposable element variation and its regulatory and epigenetic effects in the human brain
Source: bioRxiv. 2026 Jul 3:2026.07.02.735893. Preprint. [Version 1] doi: 10.64898/2026.07.02.735893 (PMC13345245; doi:10.64898/2026.07.02.735893)
Supplement: 2 [file NIHPP2026.07.02.735893v1-supplement-2.pdf]

bioRxiv preprint doi: https://doi.org/10.64898/2026.07.02.735893; this version posted July 3, 2026. The copyright holder for this preprint (which was not certified by peer review) is the author/funder. This article is a US Government work. It is not subject to copyright under 17 USC 105 and is also made available for use under a CC0 license.

Supplementary Figure Legends

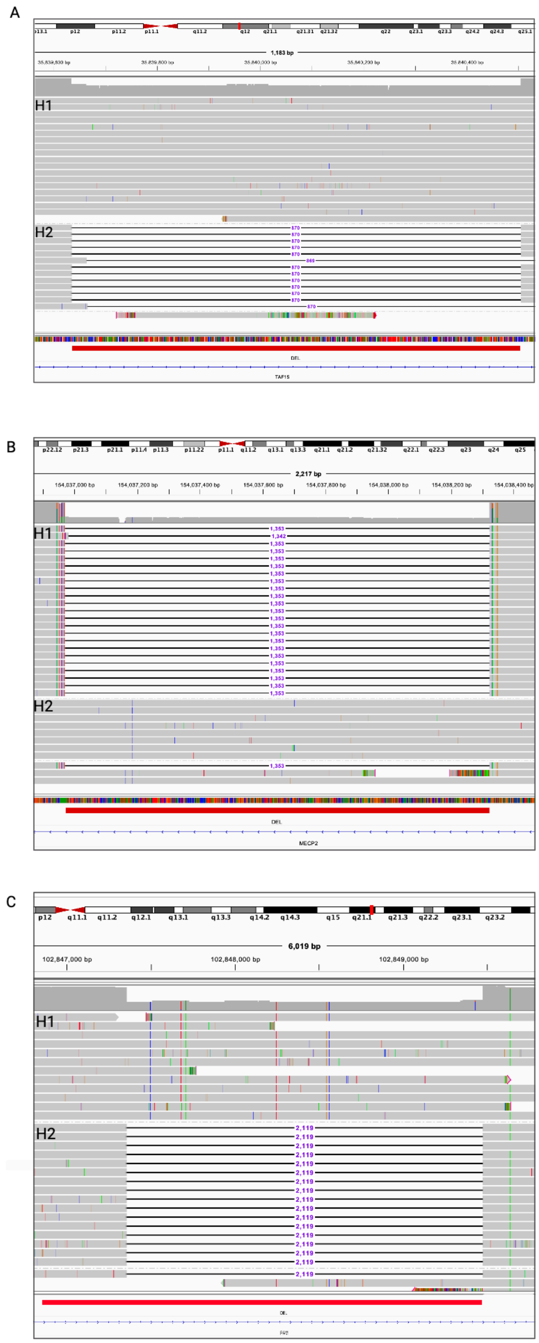

Supplementary Figure 1. **IGV Validations of TEMRs across Structural Variant Carriers.** TEMR event examples in HBCC, which overlap with (a) *TAF15*, (b) *MECP2*, and (c) *PAM* loci, were validated in IGV as heterozygous deletions within carrier samples.

bioRxiv preprint doi: https://doi.org/10.64898/2026.07.02.735893; this version posted July 3, 2026. The copyright holder for this preprint (which was not certified by peer review) is the author/funder. This article is a US Government work. It is not subject to copyright under 17 USC 105 and is also made available for use under a CC0 license.

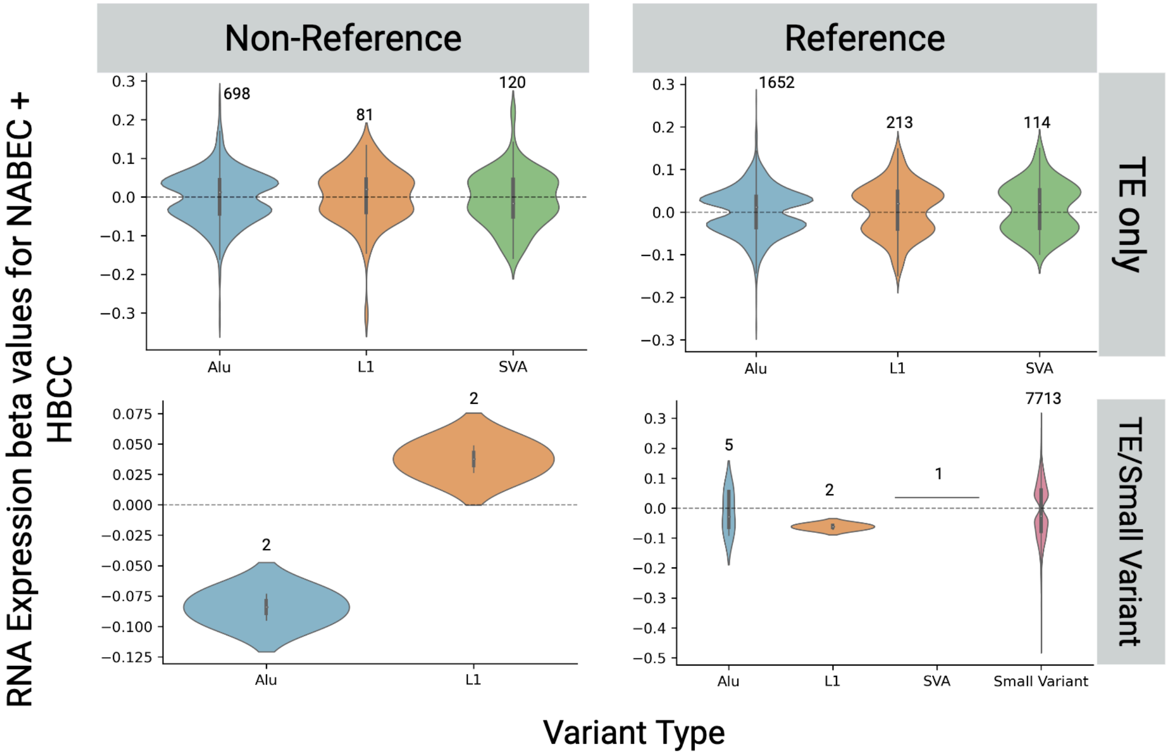

Supplementary Figure 2. Comparison of RNA expression effects by variant types. *Top Left*: TE-only eQTL by TE type for non-reference (insertions). *Top Right*: TE-only eQTL by TE type for reference (deletions). *Bottom left*: TE-Small variant joint eQTL by TE type for non-reference (insertions). *Bottom Right*: TE-Small variant joint eQTL by TE type for reference (deletions).

bioRxiv preprint doi: https://doi.org/10.64898/2026.07.02.735893; this version posted July 3, 2026. The copyright holder for this preprint (which was not certified by peer review) is the author/funder. This article is a US Government work. It is not subject to copyright under 17 USC 105 and is also made available for use under a CC0 license.

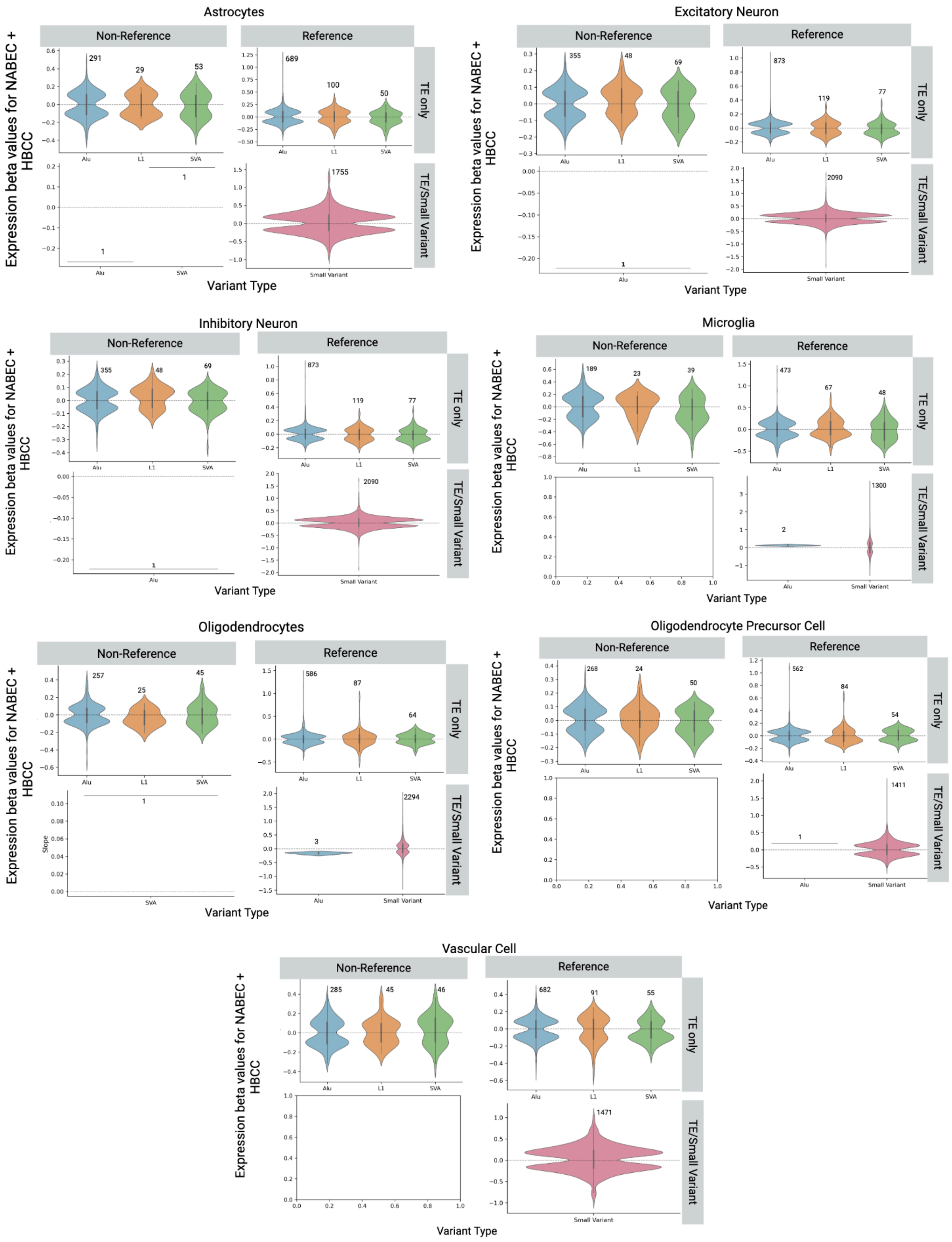

Supplementary Figure 3. Comparison of single-nucleus RNA seq expression by 7 cell types. For each panel: *Top Left*: TE-only sn-QTL by TE type for non-reference (insertions). *Top Right*: TE-only sn-QTL by TE

bioRxiv preprint doi: https://doi.org/10.64898/2026.07.02.735893; this version posted July 3, 2026. The copyright holder for this preprint (which was not certified by peer review) is the author/funder. This article is a US Government work. It is not subject to copyright under 17 USC 105 and is also made available for use under a CC0 license.

type for reference (deletions). *Bottom left*: TE-Small variant joint sn-QTL by TE type for non-reference (insertions). *Bottom Right*: TE-Small variant joint sn-QTL by TE type for reference (deletions).

bioRxiv preprint doi: https://doi.org/10.64898/2026.07.02.735893; this version posted July 3, 2026. The copyright holder for this preprint (which was not certified by peer review) is the author/funder. This article is a US Government work. It is not subject to copyright under 17 USC 105 and is also made available for use under a CC0 license.

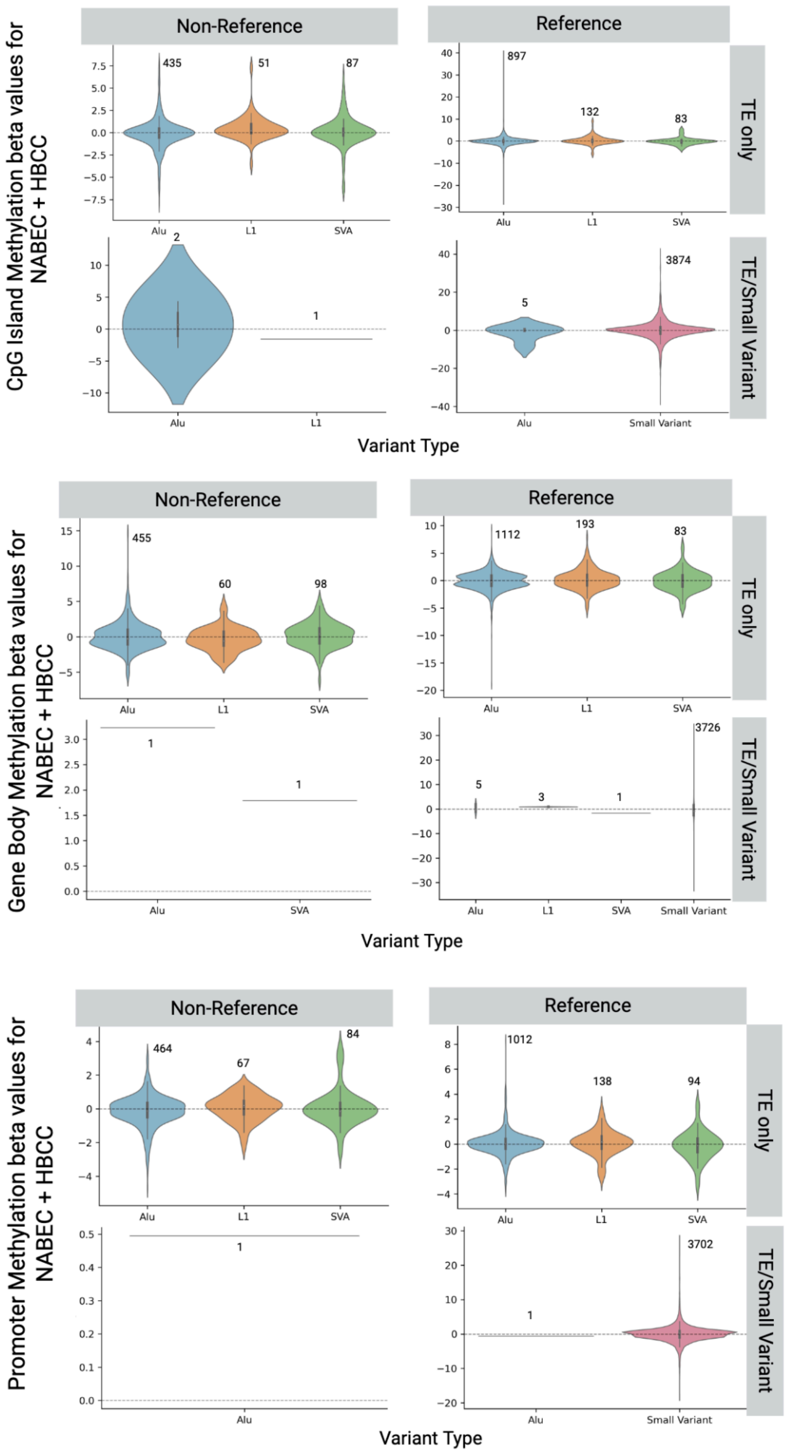

Supplementary Figure 4. Comparison of methylation effects by variant types for CGI, Promoter, and Gene Body Regions. For each panel: Top Left: TE-only mQTL by TE type for non-reference (insertions). Top Right: TE-only mQTL by TE type for reference (deletions). Bottom left: TE-Small variant joint mQTL by TE type for non-reference (insertions). Bottom Right: TE-Small variant joint mQTL by TE type for reference (deletions).

bioRxiv preprint doi: https://doi.org/10.64898/2026.07.02.735893; this version posted July 3, 2026. The copyright holder for this preprint (which was not certified by peer review) is the author/funder. This article is a US Government work. It is not subject to copyright under 17 USC 105 and is also made available for use under a CC0 license.

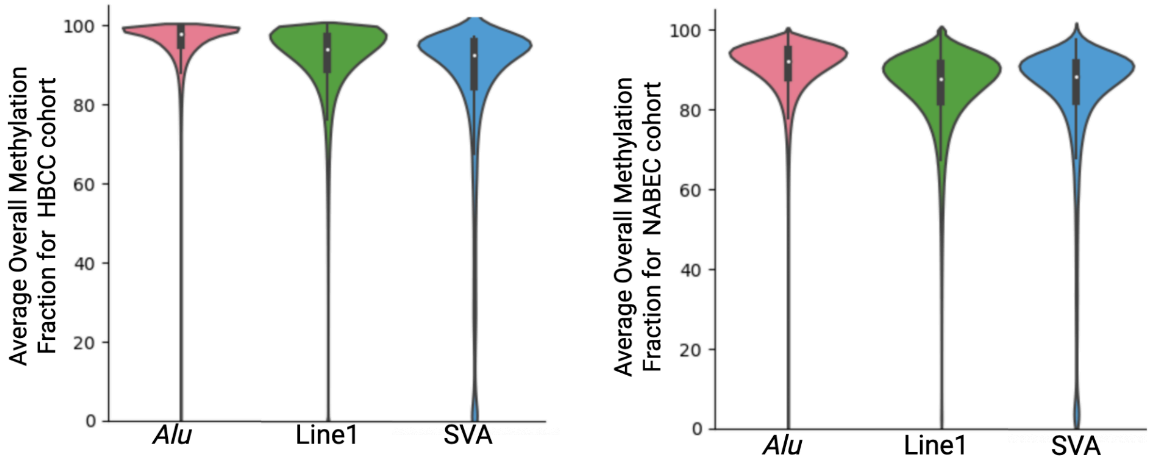

Supplementary Figure 5. Methylation patterns of *Alu*, LINE-1, and SVA elements at CpG sites in the frontal cortex. Methylation fraction (%) for both haplotypes combined, with values >80% classified as hypermethylated and <20% as hypomethylated. Left: HBCC cohort. Right: NABEC cohort.

bioRxiv preprint doi: https://doi.org/10.64898/2026.07.02.735893; this version posted July 3, 2026. The copyright holder for this preprint (which was not certified by peer review) is the author/funder. This article is a US Government work. It is not subject to copyright under 17 USC 105 and is also made available for use under a CC0 license.

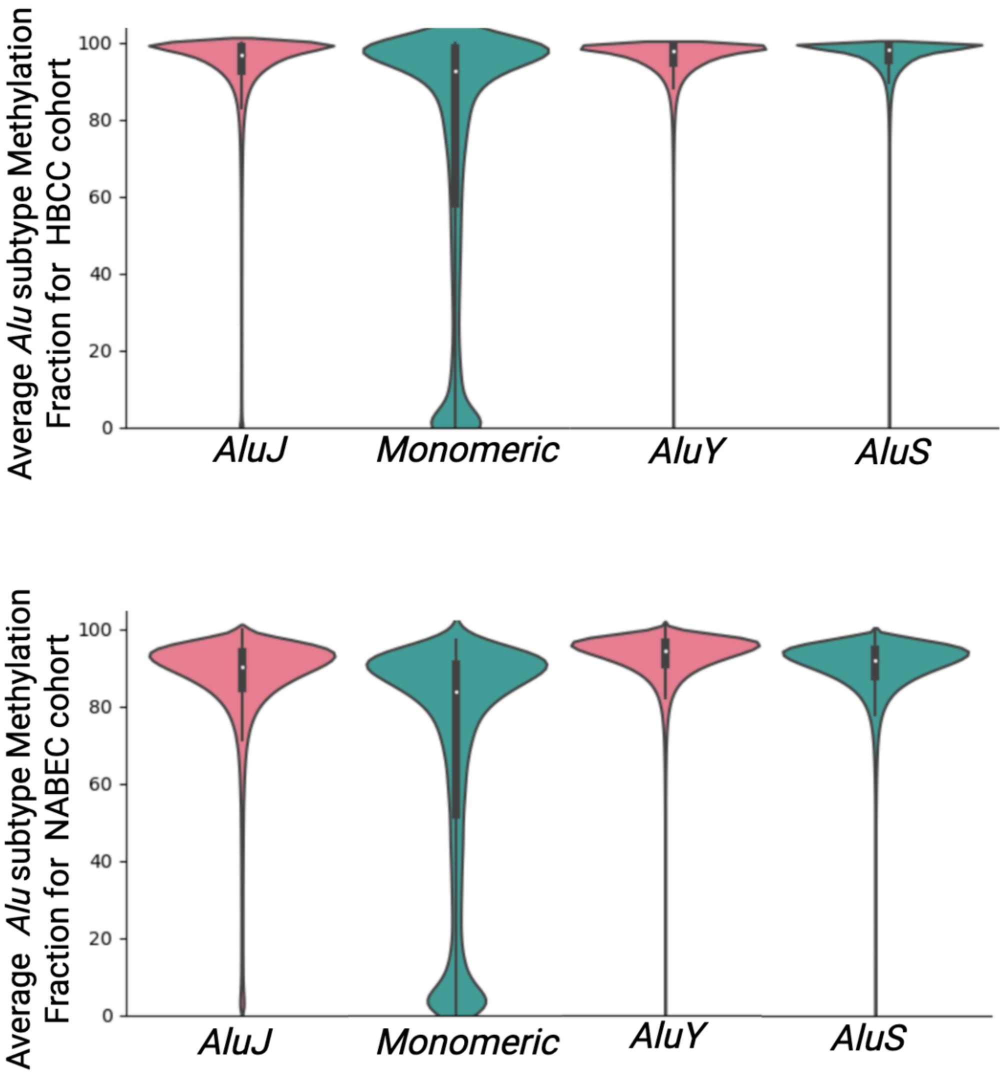

Supplementary Figure 6. *Alu* methylation patterns by subclass at CpG sites in the frontal cortex.
Alu subclasses include monomeric (oldest), *AluJ, AluS, and AluY* (youngest). **Top:** HBCC cohort. **Bottom:** NABEC cohort. Methylation fraction (%) for both haplotypes combined, with values >80% classified as hypermethylated and <20% as hypomethylated.

bioRxiv preprint doi: https://doi.org/10.64898/2026.07.02.735893; this version posted July 3, 2026. The copyright holder for this preprint (which was not certified by peer review) is the author/funder. This article is a US Government work. It is not subject to copyright under 17 USC 105 and is also made available for use under a CC0 license.

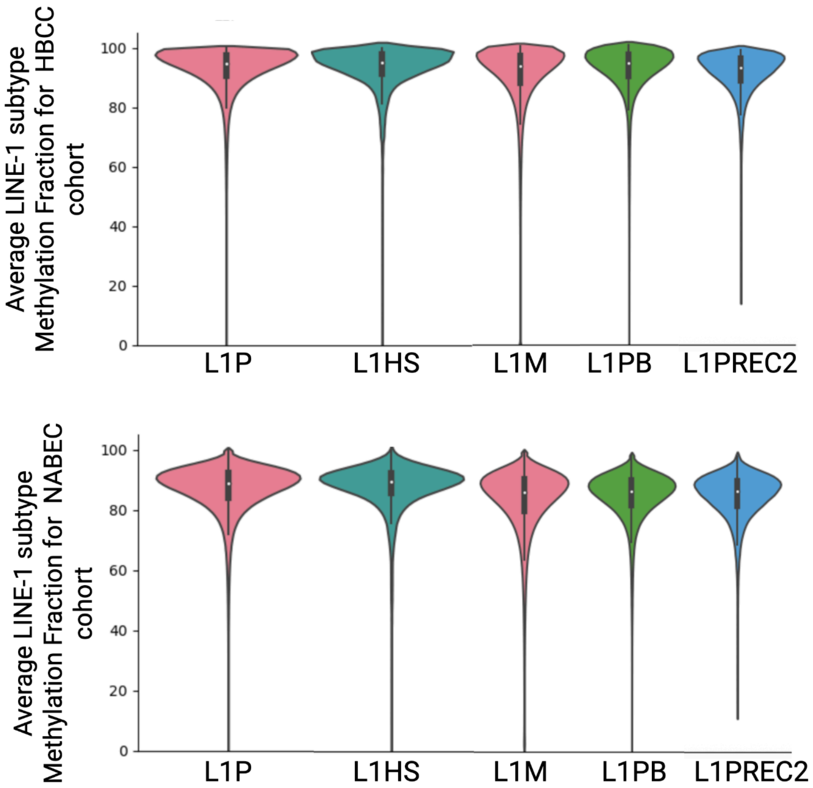

Supplementary Figure 7. LINE-1 Methylation Patterns by CpG site in the Frontal Cortex by Subclass. Classes include LINE-1P, LINE-1M, LINE-1PB, LINE-1PREC2, and LINE-1HS (the latter being the youngest and human-specific). Top: HBCC Cohort. Bottom: NABEC cohort. Methylation fraction out of 100, in which over 80 is considered hypermethylated and under 20 is hypomethylated, for both haplotypes combined.

bioRxiv preprint doi: https://doi.org/10.64898/2026.07.02.735893; this version posted July 3, 2026. The copyright holder for this preprint (which was not certified by peer review) is the author/funder. This article is a US Government work. It is not subject to copyright under 17 USC 105 and is also made available for use under a CC0 license.

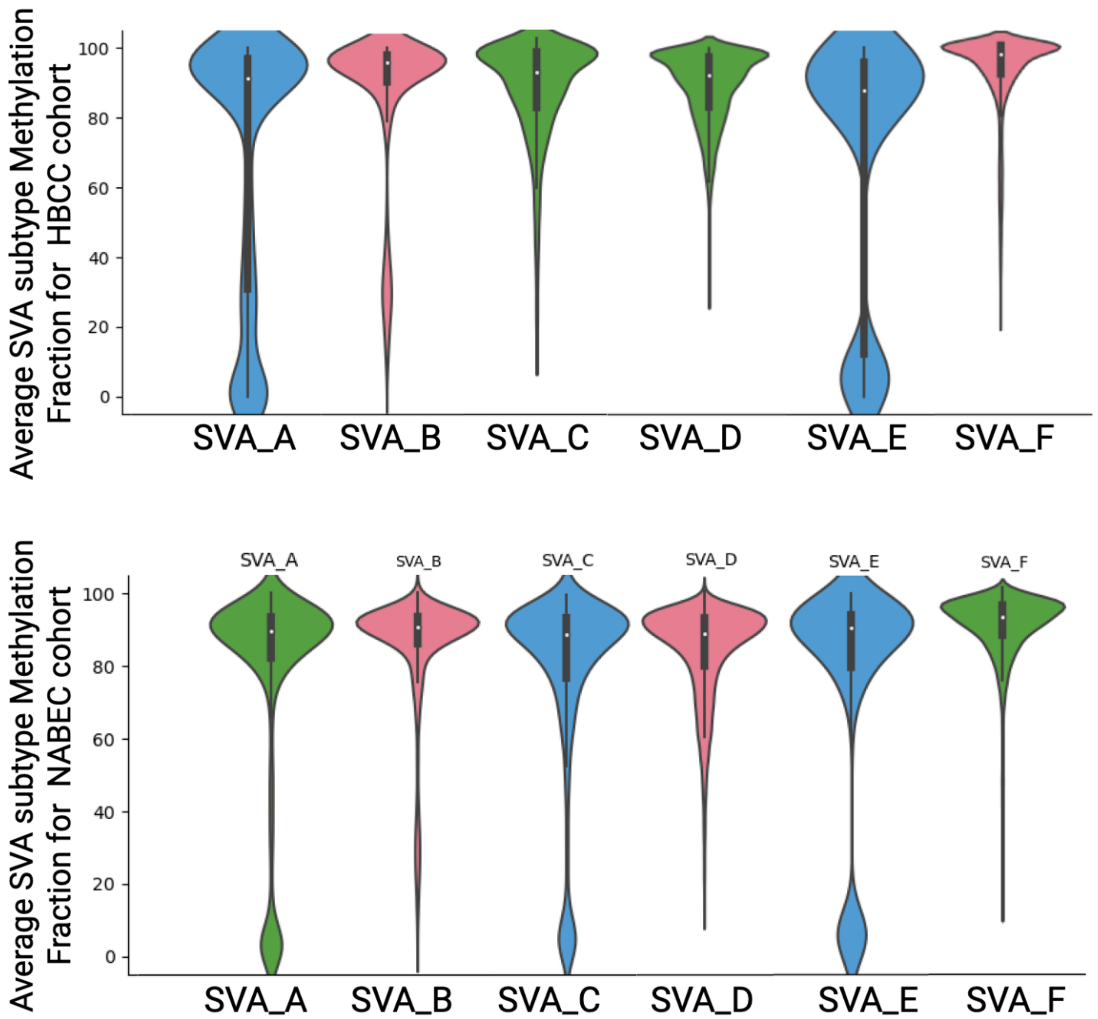

Supplementary Figure 8. SVA methylation patterns by subclass at CpG sites in the frontal cortex. Subclasses include SVA_A–SVA_F, with SVA_F being the youngest and human-specific. Top: HBCC cohort. Bottom: NABEC cohort. Methylation fraction (%) for both haplotypes combined, with values >80% classified as hypermethylated and <20% as hypomethylated.

bioRxiv preprint doi: https://doi.org/10.64898/2026.07.02.735893; this version posted July 3, 2026. The copyright holder for this preprint (which was not certified by peer review) is the author/funder. This article is a US Government work. It is not subject to copyright under 17 USC 105 and is also made available for use under a CC0 license.

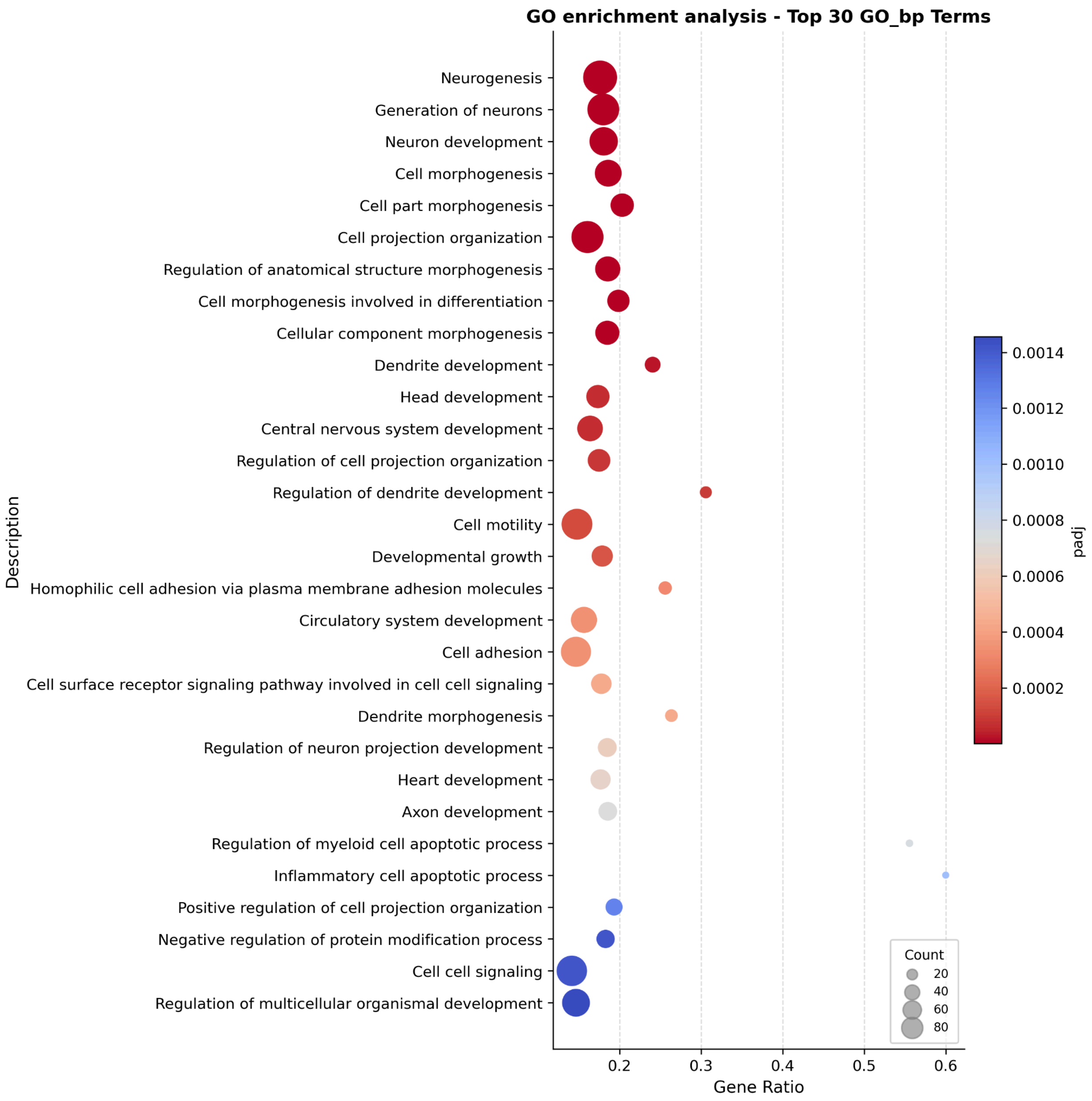

Supplementary Figure 9. GO enrichment P-values for Biological Processes associated with NABEC-specific genes. Dot size reflects how many genes are driving that enrichment; gene ratio reflects the number of genes driving enrichment relative to the total number of genes in the GO term; and dot color reflects the p-value (significance) of the enrichment.
